# An up-scaled biotechnological approach for phosphorus-depleted rye bran as animal feed

**DOI:** 10.1186/s40643-024-00765-5

**Published:** 2024-05-13

**Authors:** Niklas Widderich, Johanna Stotz, Florian Lohkamp, Christian Visscher, Ulrich Schwaneberg, Andreas Liese, Paul Bubenheim, Anna Joëlle Ruff

**Affiliations:** 1 Institute of Technical Biocatalysis, Hamburg University of Technology, Hamburg, Germany; 2 Institute of Biotechnology, RWTH Aachen University, Aachen, Germany; 3 Institute for Animal Nutrition, University of Veterinary Medicine Hanover, Foundation, Hanover, Germany

**Keywords:** Valorization of plant byproducts, Phosphorus reduced animal feed, Phosphorus mobilization, Up-scaling, Circular phosphorus bioeconomy, Phosphorus recovery, Sustainability

## Abstract

Side streams from the milling industry offer excellent nutritional properties for animal feed; yet their use is constrained by the elevated phosphorus (P) content, mainly in the form of phytate. Biotechnological P recovery fosters sustainable P management, transforming these streams into P-depleted animal feed through enzymatic hydrolysis. The enzymatic P mobilization not only enables P recovery from milling by-products but also supports the valorization of these streams into P-depleted animal feeds. Our study presents the scalability and applicability of the process and characterizes the resulting P-depleted rye bran as animal feed component. Batch mode investigations were conducted to mobilize P from 100 g to 37.1 kg of rye bran using bioreactors up to 400 L. P reductions of 89% to 92% (reducing from 12.7 g_P_/kg to 1.41–1.28 g_P_/kg) were achieved. In addition, High Performance Ion Chromatography (HPIC) analysis showed complete depletion of phytate. The successful recovery of the enzymatically mobilized P from the process wastewater by precipitation as struvite and calcium hydrogen phosphate is presented as well, achieving up to 99% removal efficiency. Our study demonstrates a versatile process that is easily adaptable, allowing for a seamless implementation on a larger scale.

**Graphical Abstract:**

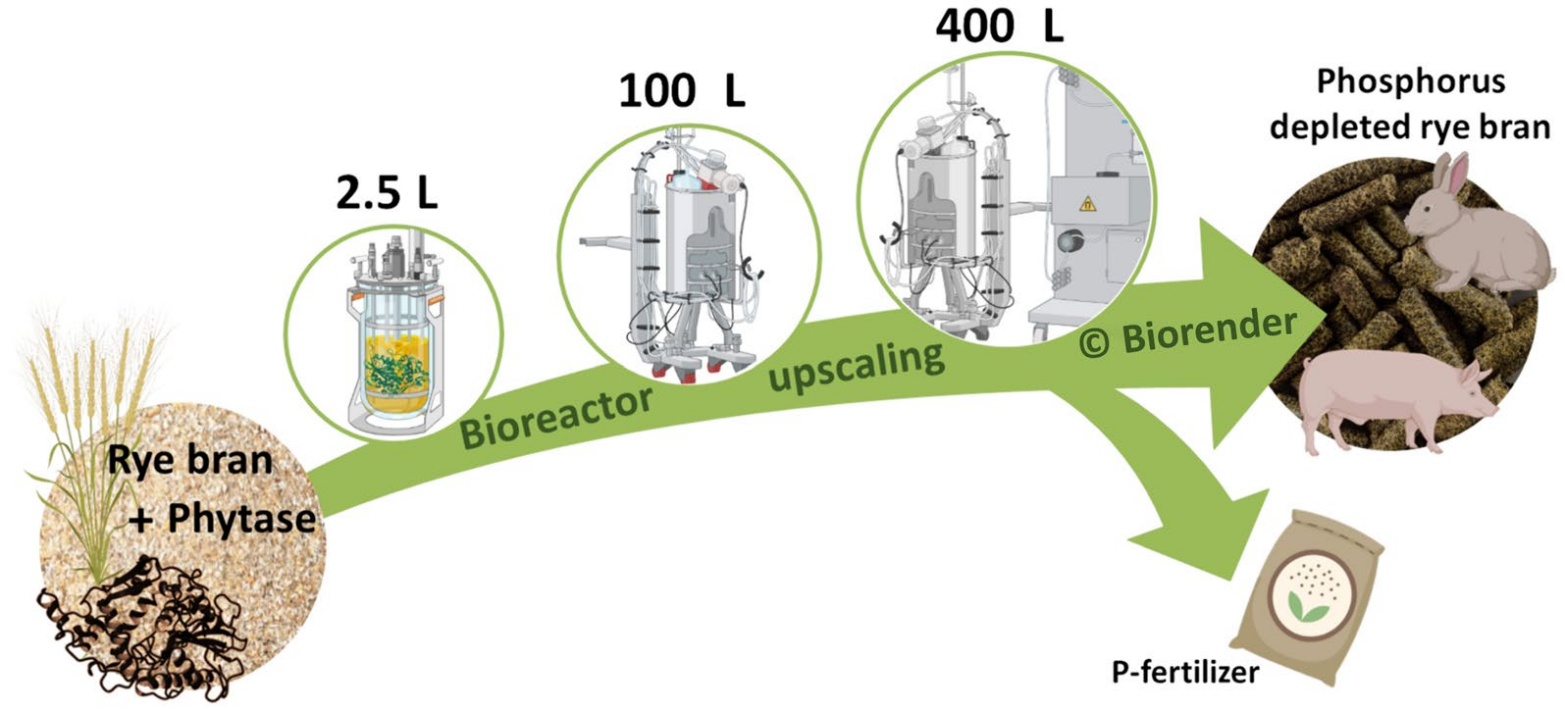

**Supplementary Information:**

The online version contains supplementary material available at 10.1186/s40643-024-00765-5.

## Introduction

Phosphorus (P) is an essential element in animal nutrition affecting productivity and growth (Sommerfeld and Rodehutscord 2019). In the main cereal-based feed components such as bran or extraction meal, the majority of P is organically bound to phytic acid (InsP_6_) and its salt (phytate) (Rosenfelder-Kuon et al. 2020), as phytate is the main P storage form in plants. It is a strong chelating agent that forms complexes with essential divalent cations, reducing the bioavailability of these minerals and making it an undesirable element in animal diets (Humer et al. 2015). Due to the limited activity of intestinal InsP_6_-hydrolyzing enzymes in monogastric animals, the phosphate cannot be completely metabolized (Hirvonen et al. 2019). As a result, feeding phytate-rich plant material leads to overfertilization, as the unused P accumulates on agricultural soils via the manure pathway. The P is then leached into water bodies, resulting in eutrophication. In consideration of increasingly stringent environmental and fertilizer legislation (DüV, StoffBilV, NEC Directive, TA-Luft) (Düngemittelverordnung 2017), nitrogen and P reduced feeding is becoming increasingly important in modern livestock nutrition (DLG Merkblatt 2021; Taube et al. 2020; DLG Merkblatt 2019).

In livestock farming, two strategies are pursued to supply P in line with the requirements. *Phase feeding* is the practice of providing feed with a lower P content that is adjusted to the animals' growth phase (Sommerfeld and Rodehutscord 2019). In the production of compound feeds for animal nutrition, the composition of the raw materials can be adjusted to provide P levels that meet the needs of the animals, reducing oversupply, subsequent P excretion and the resulting environmental impact. Feeds with high nutritional profiles such as wheat and rye bran, which are by-products of the milling industry, can only be used to a limited extend as the InsP_6_-content often exceeds 80% of the total P content. Besides phase feeding, phytase enzymes are routinely supplemented in monogastric animal diets; catalyzing the release of *ortho*-phosphate from phytate in a stepwise hydrolysis reaction (Widderich et al. 2022). However, the enzymes do not act directly on the feed but rather become active in the digestive tract (Herrmann et al. 2023). The major challenge concerning phytases in feed applications is the rapid and complete dephosphorylation of InsP_6_. The structural conformation of phytases often prevents complete hydrolysis to *myo*-inositol. Although some phytases are capable of further hydrolysing phytate to inositol-monophosphate (InsP_1_), accumulation of insP_3_/InsP_4_ is observed in the intestinal tract (Widderich et al. 2022). In addition, due to unfavorable conditions in the intestinal tract, the phytate present in the feed is only partly hydrolyzed, resulting in the excretion of up to half of the phytate taken up (Greiner and Konietzny 2006).

The finite resource P is extracted exclusively from mineral deposits, which has numerous environmental and economic drawbacks, such as heavy metal pollution and dependence on a few countries with significant deposits and exports (Rosenfelder-Kuon et al. 2020). Efficient and sustainable P recovery strategies are needed to contribute to the sustainable management of natural resources in the framework of a circular bioeconomy and thereby reducing the need for mined phosphate ores. Current research focuses on the recovery of P from wastewater using methods such as biological removal (Nielsen et al. 2019), sludge treatment (Kwapinski et al. 2021) and precipitation as struvite (González-Morales et al. 2021; Siciliano et al. 2020) or calcium hydrogen phosphate (Nguyen Quang and Ta Hong 2022; Labgairi et al. 2020).

Recently, biotechnological phosphate production from biomass has emerged as a new strategy to valorize feed material. It was first described and patented (DE 102020200670.9, DE 102018130081.6, PCT/EP2019/082709 und WO2020/109371) by the RWTH Aachen University. This P recovery strategy is a sustainable and environmentally friendly process for the production of value-added products and food additives from renewable resources. The two-step process consists of the enzymatic P mobilization of deoiled seeds or brans to completely hydrolyze InsP_6_ -rich plant material. Followed without downstream processing by a second biotransformation in which P accumulation in yeast cells leads to PolyP (polyphosphate) rich yeast extract (Herrmann et al. 2020). Laboratory-scale P recovery from various plant biomass, including bran (Herrmann et al. 2023), was shown as well as the use of bio-PolyP (bio-based polyphosphate) as a food additive demonstrated the applicability of the strategy. Thereby P-depleted feed material is prevalent.

In this study, we present the scalability of enzymatic P mobilization on the example of rye bran for the production of InsP_6_-depleted feed material and the characterization of the resulting feed. We also show for the first time the kinetics of the phytase blend used which is a combination of rPhyXT52 phytase and the phytase from *Debaryomyces castellii*, as previously described by Infanzón et al*.* (Infanzón et al. 2022). The process of enzymatic P mobilization was studied in terms of reaction conditions in a 2.5 L bioreactor and successively scaled up in different bioreactor designs up to a 400 L reaction, assessing the influence of temperature, pH control, mixing and scalability. Nutritional (P, InsP_6_, fiber, starch, fat, protein content) and physiochemical properties (water holding capacity, particle size distribution, adherent-P content) are evaluated to ensure product quality as well as consistency and are compared to the native rye bran for each process. The production of the P-depleted feed material also contributes to P recovery strategies by demonstrating that the enzymatically mobilized P can be recovered as valuable phosphate from renewable resources. This approach aims to add value to largely untapped mill industry side streams and to expand the scope of P recycling beyond the first established sources, such as wastewater treatment.

## Material and methods

All chemicals used in this study were of analytical grade or higher quality and were purchased from *Sigma-Aldrich* (Taufkirchen, Germany), *Carl Roth* (Karlsruhe, Germany) *IVA* (Meerbusch, Germany), *Bernd Kraft* (Duisburg, Germany), *Omnilab* (Bremen, Germany), *LAT* (Garbsen, Germany), *CG Chemikalien* (Laatzen, Germany) or *VWR* (Darmstadt, Germany). Rye bran was provided by *Aurora Mühlen GmbH* (Hamburg, Germany) containing a common mixture from different cultivation areas in Germany.

### Quantification of free inorganic phosphate

P quantification was performed spectrophotometrically in 96-Well microtiter plates (*Greiner, Greiner Bio-One International GmbH, Austria*) using a modified protocol of the molybdenum blue reaction according to Eeckhout and Paepe (Eeckhout and Paepe 1994). Depending on the inorganic P concentration, the sample was diluted with H_2_O to a total volume of 100 µl and then 100 µl color reagent was added. The color-developing reagent consisted of four parts of acidic 0.012 M (NH_4_)_6_Mo_7_O_24_·4 H_2_O solution and one part of 0.711 M FeSO_4_·7 H_2_O solution. After 5 min incubation, the absorbance of the formed color complex was measured at 700 nm (*Tecan Infinite® M200 pro, Tecan Trading AG, Switzerland*). For quantification of free inorganic P, a standard curve of KH_2_PO_4_ (from 50 µM to 300 µM) was used on each plate.

### HPIC analysis of inositol-phosphates

Inositol-phosphates were extracted from 5 g of biological plant material using 35 ml of 0.5 M HCl. The suspension was incubated and shaken for 16 h. Afterwards, the samples were centrifuged for 40 min, 3220×*g*, 4 °C (*Centrifuge 5804 R, Eppendorf SE, Hamburg, Germany*), and the supernatant was used for analysis. Phosphate and inositol-monophosphate to hexakisphosphates (InsP_1_ to InsP_6_) isomers were separated on a high-performance ion chromatography system (Thermo Scientific™ Dionex™ ICS-6000HPIC™ system from Thermo Fischer Scientific, Dreieich, Germany) equipped with a Dionex AXP Auxiliary Pump, Thermo Scientific Dionex EGC carbonate mixer kit, a CarboPac™ PA100 guard (4 × 50 mm) and a CarboPac™ PA100 analytical column (4 × 250 mm, Thermo Scientific). Separation was achieved using a gradient of HCl (0.5 M) followed by a postcolumn reaction in a knitted reaction coil (750 µl) with ferric nitrate (0.1% Fe(NO_3_)_3_ in 0.33 M perchloric acid) as reported in Thermo Fischer Scientific Application Note 1070 (Oates 2021). Typical retention time for PO_4_^3−^ is 5.6 min and for InsP_6_ 49.3 min. As standard phytate (Na-Phytate, Sigma-Aldrich, Steinheim, Germany) and phytic acid solution (50% (w/w), Sigma-Aldrich, Steinheim, Germany) were used. For sample preparation, the supernatant of the HCl extraction was filtered with a 0.22 µm syringe filter and two Thermo Scientific™ Dionex™ OnGuard™ II cartridges (OnGuard II RP and OnGuard II AG/H cartridge used in series).

### Analysis of feed suitability

For statements on the suitability as animal feed, common raw nutrients in the P-depleted rye bran as well as in the native rye bran were examined according to the methods of the VDLUFA (Naumann and Bassler nd). Dry matter (DM) was determined gravimetrically before and after drying the samples at 103 °C until weight constancy. The crude ash content was calculated via the mass difference after the combustion of the sample materials in a muffle furnace at 600 °C. The fiber content was determined with the Foss Fibertec 2010 Hot Extractor after washing the samples in 1.25% (v/v) H_2_SO_4_·and 1.25%(v/v) NaOH and the crude fat content was quantified by automated hydrolysis and extraction using the Hydrotherm and SOXTHERM 416 (C. Gerhardt GmbH & Co. KG, Königswinter, Germany). Crude protein contents were analyzed using a MAX N translational nitrogen and protein analyzer (Elementar Analysesysteme GmbH, 63,505 Langenselbold, Germany). The starch contents of the brans were determined polarimetrically (Schmidt und Haensch GmbH & Co., Berlin, Germany) and sugar contents were tested using the Luff-Schoorl method (Matissek et al. 1992). Additionally, calcium was analyzed by atomic absorption spectroscopy (ZEEnit 700P, Analytik Jena GmbH, Germany) and the total-P content was determined colorimetrically using a photometer (UV-1900 i, Shimadzu) at 365 nm or at NutriControl BV (Veghel, Netherland). In all approaches, the metabolizable energy (ME) in pigs was calculated according to the guidelines of the GfE (2006) taking into account analyzed values digestibility data for rye bran of the DLG (2014) (Gesellschaft für Ernährungsphysiologie 2006; Staudacher and Potthast 2014).

### Determination of physiochemical properties

Particle size distribution of dried and P-depleted as well as untreated rye bran was studied in the range of 180 µm to 2 mm by imaging (*CAMSIZER XT®, Retsch Technology, Germany*). The equivalent circle diameter was chosen for data evaluation. This corresponds to the diameter of the circle having the same projection area as the particle. X_10_, X_50,_ and X_90_ (10th, 50th, and 90th percentile respectively) were derived from the volume-specific particle size distribution. For further investigation, the flour fraction (180, 200, 224 µm mesh size) was sieved off (*Retsch AS200 Control, Retsch Technology, Germany*) for 5 min at 60% of the maximum amplitude in the native substrate. The percentage by mass of flour was determined gravimetrically.

Water holding capacity (WHC) was determined using a modified protocol of Petersson et al. (2013). Five g of biological material was incubated in 35 ml H_2_O and shaken at 120 rpm. After 30 min of incubation, the bran/water slurries were centrifuged at 3860×*g* for 20 min (*Universal 320 R, Andreas Hettich GmBH & Co. KG, Tuttlingen, Germany*). The supernatant was carefully removed and the centrifuge tube containing the pellet and adsorbed water was weighted. The WHC was calculated as g water g^−1^ original dry sample.

The adherent P content after enzymatic P mobilization and drying was determined. Five g of the conditioned rye bran and 40 ml H_2_O were shaken in a centrifuge tube at 200 rpm. After 20 min the suspension was centrifuged at 3860×*g* for 20 min. The P content in the supernatant was determined by the molybdenum blue color reaction and correlated with the amount of plant material.

### Production of phytase

Expression of rPhyXT52 phytase (Tan et al. 2016) and *Debaryomyces castellii* phytase (GenBank accession numbers KM873028, EF121003) (Ragon et al. 2008) in *Pichia pastor*is BSYBG11 using the plasmid pBSY3S1Z was performed as described in Infanzon et al*.* (2022). Flask expressions of phytase genes under the control of the CAT promoter (BSY3S1Z plasmids) were supplemented with 150 µg/ml Zeocin. Preculture (15 ml, 48h, 30 °C, 250 rpm) was performed in YPD medium (2% peptone, 1% Bacto yeast extract from BD Biosciences (Miami, USA), 2% Glucose sterilized separately). For cultivation of the main culture, an adapted protocol was used (Hartner et al. 2008): Main culture of 185 ml BMD medium (200 mM potassium phosphate buffer at pH 6, 1.34% (w/v) yeast nitrogen base without amino acids (Formedium, Norfolk, England), 1% (w/v) glucose and 4·10^–5^% (w/v) d-biotin; Higher concentrated stock solutions of all components were separately prepared in deionized H_2_O (dH_2_O) and sterilized) was inoculated with 6.1 ml pre-culture in a 2 L Erlenmeyer flask and grown for 48 h (30 °C, 250 rpm). Induction was performed by the addition of 153 ml BMDM-1 (BMD medium supplemented with 1% (v/v) methanol) and followed by further incubation for 72 h. Every 24 h additional methanol was supplemented by adding 30 ml of BMDM-5 (BMD medium with 5% (v/v) methanol). The culture supernatant was collected by centrifugation (4 °C, 3220×*g*, 60 min Sorval RC 6 + centrifuge, Thermo Fisher scientific, Waltham, USA) and stored at 4 °C.

### Kinetic investigation of phytase blend

Kinetic studies of the phytase blend were conducted in a thermoshaker (T_S1_ Thermoshaker, Biometra Biomedizinische Analytik GmbH, Jena, Germany) at 37 °C by varying the concentration of phytate (P8810, Sigma-Aldrich, Taufkirchen, Germany) from 0.1 mM to 15 mM in a reaction solution (50 mM NaOAc, pH 4.4). The distribution of differently phosphorylated inositols in the phytate standard corresponds to their distribution in rye bran (Fig. 3). The reaction was started by addition 20 µl of cell-free extract in appropriate dilutions. Each reaction batch had a total volume of 1 ml. In order to not exceed a conversion of 10%, the added volume of cell-free enzyme extract was reduced from 0.01 ml to 0.001 ml in appropriate dilutions with reduced substrate concentration. In addition, the reaction was stopped after 3 min using 10% (w/v) TCA. The liberated inorganic P was determined by the molybdenum blue color reaction described above. Thus, one U is defined as 1 µmol of inorganic P released in 1 min at 37 °C and pH 4.4 at varying phytate concentrations. Nonlinear fitting of the data was performed in Origin 2022 software (OriginLab, Northampton, USA) using the Michaelis–Menten equation.

### Phosphorus mobilization from biomass

Phosphate release from biomass was performed as previously described in Herrmann et al. (2020). In summary, the bran was resuspended in phytase reaction buffer (50 mM NaOAc pH 5.0) using a ratio of 1 to 7 in a 5 L beaker with a lid. The reaction was started by adding a phytase blend of rPhyXT52 phytase (Tan et al. 2016) and *Debaryomyces castellii* phytase (Oates 2021) in a 1:1 ratio (based on activity for InsP_6_). A total of 4000 phytase units (2000 U rPhyXT52 phytase + 2000 U *Debaryomyces castellii* phytase) per kg bran were used. After incubation for 12 h (37 °C, 180 rpm), the phosphate-containing supernatant was separated from the biomass by centrifugation (4 °C, 3220×*g*, 20 min; Eppendorf Centrifuge 5810 R, Hamburg, Germany) and decantation. The Bran pellet was dried (48 h, 65 °C in Binder FP Drying Chambers with forced convection, Binder, Tuttlingen, Germany) for further analysis.

P mobilization on large scale was performed in a 400 L bioreactor (PID 2 media preparation vessel, Bioengineering, Wald, Switzerland) equipped with a 3-winged propeller impeller (ø 150 mm) filled with 37.1 kg of bran and 248 L tab water as well as in a 100 L bioreactor (Laboratory Pilot Fermenter LP351; Bioengineering, Wald, Switzerland) filled with 8.6 kg of bran and 65 L of deionized water and in a 2.5 L bioreactor equipped with two stirring blades (Minifors, Infors HT, Bottmingen, Switzerland) filled with 200 g of bran and either 1.2 L of tap water, deionized water or buffer (50 mM sodium acetate pH 5.5). pH was adjusted to 4.4 with 2 M HCl in case of water and after the mixture reached temperature, phytase was added (rPhyXT52 phytase, and Debaryomyces castellii phytase; 4000 U per kg each) and the reaction was carried out under stirring (19 h at 910 rpm in 400 L scale; 6 h at 500 rpm in 100 L scale; 12 h at 700 rpm in 2.5 L scale). The stirrer speed in the bioreactors was comparable but settled different in rpm due to different conformation of the blades and vessel size. Due to economic feasibility, the temperature of the largest scale production (400 L reaction) was not controlled. pH control in bioreactors was done with NaOH and HCl (2 M in 2.5 L and 65 L scale; 5 M in 400 L scale). Supernatant and biomass were separated by sedimentation, biomass was washed three times with tap water in either half or equal volume as reaction volume (122 L, 65 L, or 1.2 L respectively), and separation was performed by sedimentation between each washing step. In case of 400 L and 65 L scale, final separation was achieved by using a 10 L stainless steel screw press equipped with a press bag (Paul Arauner GmbH & Co. KG, Kitzingen, Germany). Washed bran pellets were dried to a dry matter content of more than 90% by either lyophilization (Christ Alpha 1–4, Martin Christ GmbH, Osterode, Germany) (400 L scale) or 48 h to 72 h at 65 °C in a drying chamber (Binder FP Drying Chambers with forced convection, Binder, Tuttlingen, Germany) to increase shelf life for further use.

### Phosphate precipitation

Precipitation was used for P recovery in the process wastewater; that is, from the supernatant after the initial sedimentation of the 400 L reaction. The effluent was centrifuged at 10,900×*g* (Avanti J-25, Beckman Coulter Life Sciences, Krefeld, Germany) for 1 h to sediment the remaining organic material. 60 ml of the supernatant containing the liberated P was used for precipitation experiments. The experimental setup consisted of a 100 ml beaker with a magnetic stirrer and a pH meter. The temperature was not controlled; i.e. room temperature. Stoichiometric amounts of MgCl_2_ and NH_4_Cl, corresponding to the inorganic P concentration, were used for struvite precipitation. The pH was adjusted to pH 9 with 1 M NaOH and the reaction time was 1 h. Similarly, a stoichiometric amount of Ca(OH)_2_ was used for calcium hydrogen phosphate precipitation. Due to its low solubility, Ca(OH)_2_ was added gradually over a period of 45 min. The reaction was allowed to continue for 16 h, with the pH being monitored but not controlled. To determine the phosphate precipitation yield, the inorganic P concentration was measured before precipitation and after filtration (*Fisherbrand QL 115 filters*, *Thermo Fisher Scientific, Darmstadt, Germany*) of the precipitates in the filtrate. Taking into account the P precipitation yield and the molar mass of the precipitates, the theoretical mass of the precipitate to be obtained was calculated. The purity was determined by relating the theoretical mass to the actual mass obtained.

### XRD analysis of the harvested precipitates

The precipitates were filtered (*Fisherbrand QL 115 filters*, *Thermo Fisher Scientific, Darmstadt, Germany*) and dried at 40°C for 48 h before X-ray diffraction (XRD) analysis. A Siemens D500 (*Siemens, Munich, Germany*) was used for XRD measurements, which were performed at room temperature using Cu Kα radiation. The XRD patterns were recorded in the 2 θ scanning range from 3° to 63° every 0.05° at a resolution of 1 s per increment.

## Results and discussion

The hydrolysis of phytate is evaluated at different P mobilization reaction scales and conditions and the efficiency is compared. A comparison between the native rye bran and the conditioned one is made at all process scales. This is necessary because changes in physiochemical and nutrient properties of rye bran affect its functionality and applicability in feed formulations. P recovery from the process wastewater (liquid phase containing mobilized P) for fertilizer applications is assessed as well by investigating two different precipitation strategies.

### Phosphorus mobilization

**Fig. 1:**
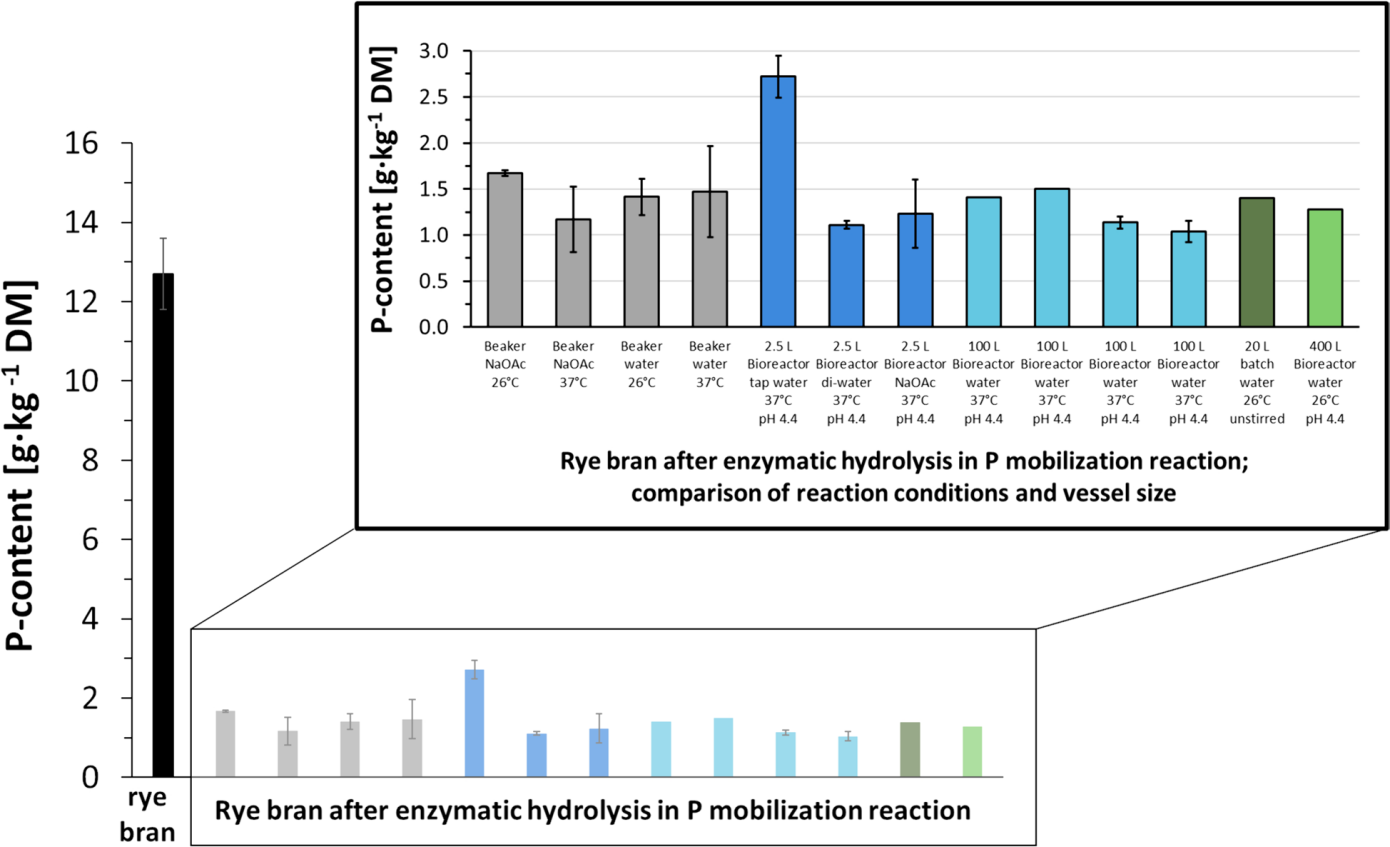
Total P content of rye bran and Rye bran after P mobilization in different reaction setup up. Rye bran was enzymatically treated in bioreactor vessels and the total P content was determined by ICP-OES. See Table S1 and material and method section for detailed parameters of reaction conditions. 4000 units of phytase blend of rPhyXT52 phytase (Tan et al. 2016) and Debaryomyces castellii phytase (Oates 2021) per kg bran, pH 4.4 (buffered or titrated), 37 °C or 26 °C

Enzymatic mobilization of P from rye bran in an aqueous medium is performed in bioreactor vessels of 2.5 L to 400 L. Reaction conditions were adapted from previous studies reporting P mobilization from deoiled seeds and bran in small-scale reactions in which 5 g up to 400 g were processed (Infanzón et al. 2022). First, in a 2.5 L bioreactor with 200 g rye bran, reaction conditions like temperature (26 °C, 37 °C), pH influence (pH maintained at 4.4), and reaction medium (buffer, water, tap water) were investigated. For an economical process design, the conditions for the P mobilization were set to water as reaction medium, a temperature of 26 °C and pH regulation to pH 4.4. Then the process was scaled up in 100 L and 400 L bioreactors in which 8.5 kg and up to 37.1 kg bran were processed respectively. In total 46 samples were analyzed to determine the total P content of the performed enzymatic P mobilization reactions (four reactions in 0.7 L scale, five reactions in 2.5 L as well as four reactions in 100 L and one on 400 L scale). The latter are listed in Table S1 in Supplementary. The total P content, the obtained P from the reduction of the treated bran as well as the detailed reaction parameters (Table S1) are summarized graphically in Fig. 1. As reference native rye bran without any treatment was analyzed.

We could demonstrate that the total P content could be reduced on average by 88.7% from 12.7 g_P_/kg rye bran to 1.43 g_P_/kg by enzymatic hydrolysis combined with soaking in aqueous medium. Reaction conditions close to the optimal reaction conditions of the added phytases are the most efficient, leading to a P depletion of 91.2% in rye bran and reached a remaining P content of only 1.11 gP/kg. An increased P mobilization of 42% can be reached by increasing the temperature from 26 °C to 37 °C and using NaOAc as the reaction medium (1.17 g/kg rye bran compared to 1.67 g/kg). In contrast, no difference was observed at 26 °C or 37 °C, when water without pH titration was used as the reaction medium. A difference of more than 2.45 times lower P content was observed in the 2.5 L scale when comparing water to tap water and buffer at 37 °C with pH maintained at 4.4 (tap water 2.72; deionized water 1.11; buffer 1.23 g_P_/kg rye bran). Nevertheless, we concluded that the process can be conducted in a wide range of reaction parameters. The differences in the P reduction rate (1.43 g_P_/kg rye bran ± 0.28 g_P_/kg) result from the natural occurring variation of phosphate content in the starting material/ rye bran (12.7 g_P_/kg rye bran ± 0.9 g_P_/kg). Furthermore, reaction parameters in 400 L scale-up at 26 °C and water with pH titration to 4.4 are as efficient as in 100 L scale-up and reached a total P reduction of 90% (12.7 to 1.28 g_P_/kg rye bran).

**Fig. 2:**
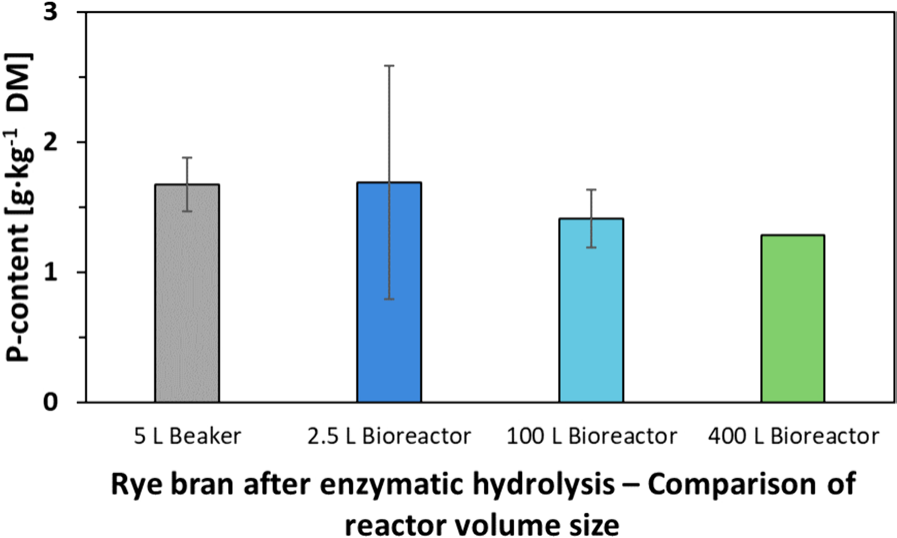
Rye bran after enzymatic hydrolysis—Comparison of reactor size in P mobilization reaction. Reaction conditions: 4000 units of phytase blend of rPhyXT52 phytase (Tan et al. 2016) and Debaryomyces castellii phytase (Oates 2021) per kg bran; pH adjusted to 4.4 with NaOH and HCl; 400 L scale tap water 19 h at 910 rpm; 100 L scale deionized water 6 h at 500 rpm; 2.5 L scale 12 h at 700 rpm in buffer or water

Indeed, the vessel size did not impact the process (Fig. 2). A scale-up from 100 g to 37 kg rye bran in 0.7 L to 400 L reaction volume scale was successful and reached a P reduction of up to 89% to 92% (reduction from 12.7 g_P_/kg to 1.41–1.28 g_P_/kg). Similar P reduction rates were reported for rape or sunflower meal (82% to 90%), where the process was previously performed on laboratory scale only (Herrmann et al. 2023; Infanzón et al. 2022).

**Fig. 3:**
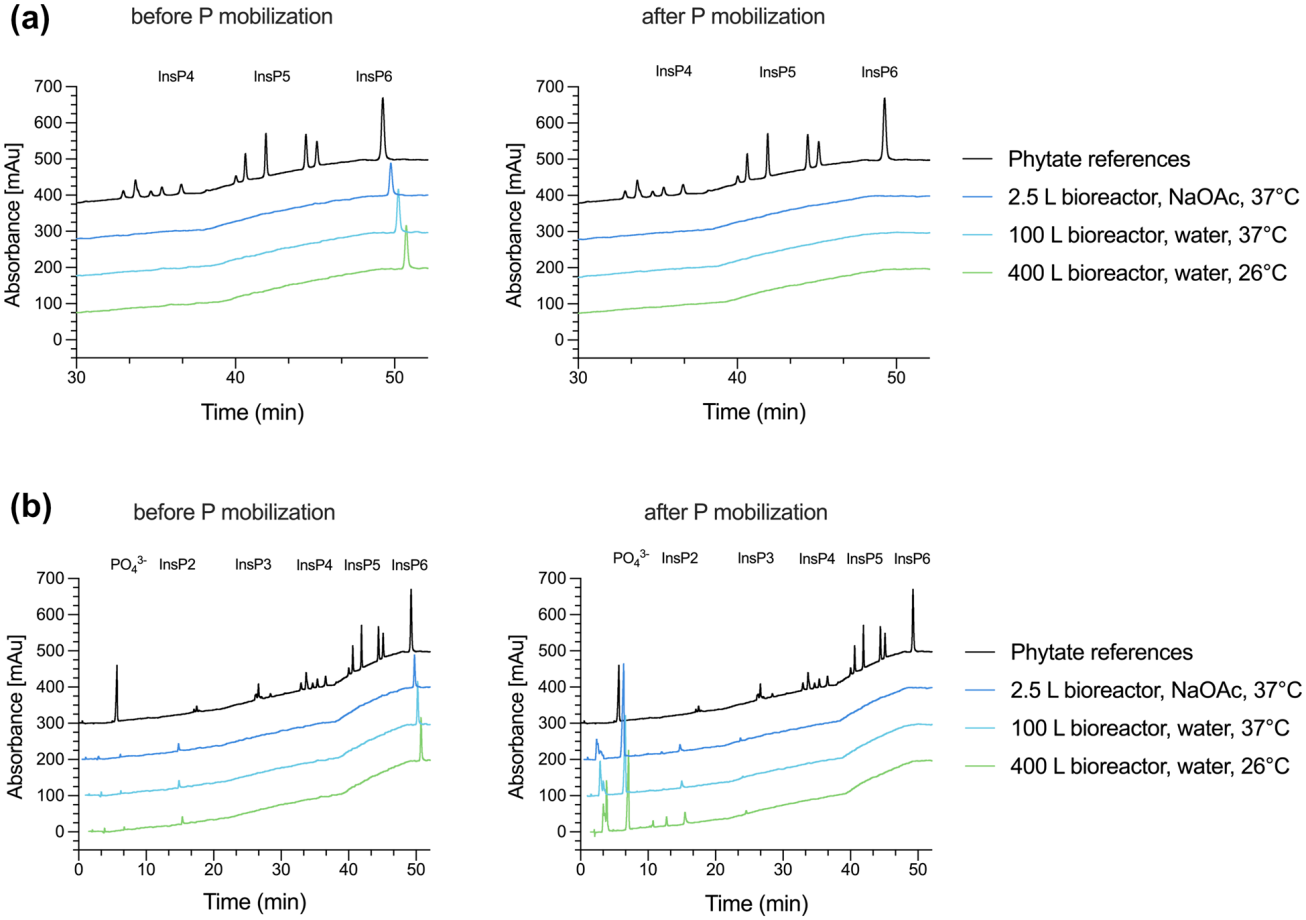
HPIC analysis of InsP_6_ in HCl-extracted rye bran before and after the P mobilization process comparing different reactor sizes. **a** HPIC chromatogram extract from 30 to 50 min. **b** HPIC chromatogram from 0 to 50 min. InsP_6_ (monitored after at 49.3 min after injection) could not be detected after the P-mobilization process and increase of PO_4_^3−^ could be detected (peak monitored at 5.6 min after injection). Additional HPIC chromatograms of InsP6, PO_4_^3−^ standards and phytate references are to be found in the supplementary Figure S1 and S2

Stirring was crucial to increase mass transport, as without stirring (unstirred 20 L batch reaction Nr. 12 in Table S1) an incomplete InsP_6_ hydrolysis was obtained. In non-stirred systems, a small remaining InsP_6_ content of around 8% InsP_6_-content of native rye bran and small amounts of InsP_5_ was identified by HPIC analytic (Fig. 3, InsP_6_ peak at around 50 min after injection).

Washing the biomass after P mobilization impacts the remaining P content, as the concentration of inorganic P in the supernatant determines the adherent P content after drying and thus the total P content of the feed material produced (Tables 1 and 2). On average the P content in the supernatant is 1.70 g PO_4_^3−^/L, depending on the biomass-to-liquid ratio (1:7 w/v). Nevertheless, after sedimentation and a single wash, the P content is reduced by 47% to 0.90 g PO_4_^3−^/L. After two and three washes, the P content further decreases to 0.55 g PO_4_^3−^/L and 0.16 g PO_4_^3−^/L, respectively, resulting in a 92% reduction of inorganic P content in the supernatant.

**Table 1:** Comparison of nutrient properties between native and P-depleted bran at various 100 L and 400 L production scales

	Unit	Native rye bran	Treated rye bran				
Reactor volume	L		100	100	100	100	400
Dry matter	g/kg oS	902.0	971.0	970.0	977.0	977.0	980.0
Crude ash	g/kg DM	63.0	7.0	8.0	7.3	5.7	6.2
Crude protein	g/kg DM	165.7	124.9	96.5	114.1	118.8	99.6
Crude fat	g/kg DM	32.7	46.7	43.0	50.1	50.3	54.9
Crude fiber	g/kg DM	65.2	121.3	122.4	127.4	121.3	132.8
Starch	g/kg DM	151.9	66.1	87.3	78.3	74.8	104.6
Sugar	g/kg DM	108.2	9.3	8.5	9.9	9.3	0.9
Ca	g/kg DM	< 1	1.6	1.5	< 1	< 1	< 1
P	g/kg DM	12.9	1.4	1.5	1.1	1.1	1.3
ME_Pig_	MJ/kg DM	10.9	11.0	10.9	11.0	11.1	11.1

oS: origin substance; DM: Dry matter; ME_Pig_: metabolizable energy, calculated for pigs according to the guidelines of the GfE (2006) (Gesellschaft für Ernährungsphysiologie 2006)

**Table 2:** Comparison of physiochemical properties before and after P mobilization at different production scales. Duplicates are given to show biological fluctuations

	Unit	Native	Treated rye bran						
Reactor volume	L	/	2.5 water (1)	2.5 water (2)	2.5 buffer (1)	2.5 buffer (2)	100 (1)	100 (2)	400
X_10_	µm	432.7 ± 9.3	357.6 ± 21.9	401.2 ± 41.3	389.3 ± 45.1	317.1 ± 0.0	409.6 ± 22.7	393.7 ± 19.8	271.5 ± 3.7
X_50_	µm	788.8 ± 36.7	915 ± 102.1	1047.9 ± 131.7	980.5 ± 184.4	883.1 ± 0.0	816.6 ± 49.7	798.5 ± 52.7	572.2 ± 7.0
X_90_	µm	1448.0 ± 223.0	2126.2 ± 119.0	2175.4 ± 179.8	2008.1 ± 149.3	1933.5 ± 0.0	1727.6 ± 84.4	1705.9 ± 64.7	1417 ± 50.1
WHC	ml/g_DM_	2.8 ± 0.1	5.1 ± 0.3	5.8 ± 0.2	4.8 ± 0.2	5.1 ± 0.2	5.0 ± 0.3	5.1 ± 0.2	5.3 ± 0.4
Adherent P	g_P_/kg_DM_	/	0.7 ± 0.1	0.7 ± 0.0	1.0 ± 0.0	0.9 ± 0.0	0.4 ± 0.0	0.5 ± 0.1	0.4 ± 0.0

(1) and (2) each refer to biological duplicates. 2.5 L water (1): tap water; 2.5 L water (2): deionized water, pH titration to 4.4; 2.5 L (1) buffer: 50 mM NaOAc pH 5.0; 2.5 L (2) buffer: 50 mM NaOAc pH 5.0; 100 L (1): deionized water, pH titration to 4.4, 100 L (2): deionized water, pH titration to pH 4.4; 400 L: tap water, pH titration to 4.4

The method used for separating rye bran from the liquid phase has an impact on the recovery of bran. Bran weight loss of up to 40% was observed. Particle content for sizes smaller than 180, 200, and 220 µm being 10.0, 14.9, and 20.1 wt.-%, respectively. The first fraction consists of only white flour, while the second fraction contains darker particles, and the third fraction contains very small bran particles. Therefore, it is concluded that the loss of small-size particle fractions already causes a large weight loss. Finely grounded material and remaining flour is washed out and cannot be retained when decantation and pressing through filters is applied, as reflected in the equivalent circle diameter at X_10_ (Table 2).

HPIC analysis was performed with HCl-extracts of rye bran before and after the P mobilization process to determine the hydrolysis of InsP_6_ and the lower inositol-phosphates content. The InsP_6_-content of the P mobilization reactions is reduced by 92% for the unstirred system and converted fully to phosphate in all stirred systems, regardless of the bioreactor size and reaction volume (Fig. 3).

**Fig. 4:**
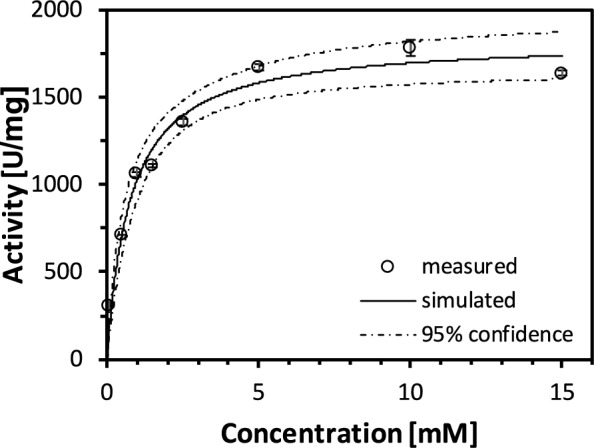
Kinetics of rPhyXT52 and *D. castelli* phytase cell-free extract blend at 37 °C in 50 mM NaOAc buffer pH 4.4. Both enzymes are individually expressed in *P. pastoris*

Kinetic investigation of the phytase blend used for P mobilization shows no clear inhibition by substrate or products (*ortho*-phosphate, partially phosphorylated inositols, *myo*-inositol) in the concentration range considered (up to 15 mM). For *D. castelii* phytase, however, inhibition by phosphates has been demonstrated at substrate concentrations lower than 8 mM (Ragon et al. 2008). Nevertheless, there is a slight reduction in activity at a substrate concentration of 15 mM, as shown in Fig. 4. However, this is considered to be an outlier. In addition, the concentration of inositol-phosphates that are transferred to the liquid phase is always at a low level due to the ongoing hydrolysis. Only the accumulating *ortho*-phosphate could have led to a small inhibitory effect. The hydrolysis is catalyzed as a double substrate kinetic. However, since H_2_O is present in excess in the aqueous system and can therefore be neglected, the phytase blend follows a simple Michaelis Menten kinetics as expected (Fig. 4). Simulation to the measured data has a correlation coefficient *R*^*2*^ of 0.97, with all measured data within the 95% confidence level. The concentration range investigated was chosen to cover the maximum possible concentration of inositol-phosphates in the liquid phase. Therefore, the phytase blend is suitable for batch operation at a given bran-to-liquid ratio of 1:7 w/v. Curve fitting to the measurement data gives a Michaelis–Menten constant K_M_ of 0.78 ± 0.12 mM and a maximum specific reaction rate v_max_ of 1828 ± 68 U/mg. Compared to literature data for the individual enzymes (*D. castellii*, expressed in *P. pastoris*, 182 U/mg (in 200 mM NaOAc, pH 4, 37 °C, 8 mM sodium phytate) (Ragon et al. 2008) rPhyXT52 phytase *fungus garden metagenome*, expressed in *E. coli*, 3102 U/mg (in 200 mM acetate buffer, pH 3.9, 37 °C, 1 to 5 mM phytate) (Tan et al. 2016)), the v_max_ value lies in the arithmetic mean. Whereas the K_M_ value (*D. castellii* (Ragon et al. 2008)) is slightly elevated but can be attributed to differences in pH, salt concentration, substrate range, and the substrate itself. In this study, a mixture of differently phosphorylated inositol-phosphates was used, similar to the distribution in rye bran. The HPIC chromatogram for the P mobilization process shows no peak for InsP_6_ and InsP_5_ at 50 min (Fig. 3). Even further, no lower inositol-phosphates (InsP_2_ to InsP_5_) and no InsP_1_ could be detected by HPIC, but only phosphate (peak monitored at 5 min after injection, Fig. 3b) in treated bran.

From the results we conclude that the P mobilization process reaches the complete hydrolysis of InsP_6_. The obtained treated rye bran does not contain any residual InsP_1_ to InsP_6_ and is therefore a valorized feed material. Phytate-reduced feed material is aimed to decrease the indigestible phytate content, to decrease the P excretion in manure and consequently the environmental burden. Furthermore, the process exhibits adaptability to various reaction parameters, which allows flexibility in its practical implementation and thus rendering the process more economical.

### Nutrient properties before and after conditioning

The results of the feed analysis of native and treated rye bran are shown in Table 1. Lower crude ash and crude protein contents and higher crude fat and crude fiber contents were determined in the treated rye brans compared to the native rye bran. The amounts of carbohydrates starch and sugar were also lower (Table 1). The treatment of the native rye bran reduced the total P content by 88% to 92%. Furthermore, regardless of the reactor size, P mobilization does not influence the calculated energy values.

The treatment of the native rye bran led to changes in the nutrient composition, which can be explained by the P mobilization process. The process conditions in the present study increased not only the solubility of P but also the solubility of other nutrients. Mainly due to the loss of crude ash, starch, water-soluble proteins, and other soluble polysaccharides during washing, the fiber and fat content increased. Similar findings have been observed by Nyombaire and Ng (Nyombaire and Ng 2022). Furthermore, it is known from other investigations that native rye grain contains both endogenous enzymes and indigenous microbes in the outer layers of the grain, which themselves also generate hydrolytic enzyme activities and can lead to altered grain composition after fermentative processes (Brijs et al. 1999; Loponen et al. 2004; Katina et al. 2007). The extraction of protein from bran depends on conditions during treatment, such as pH, time, temperature, solvent, and particle size (Roberts et al. 1985). The only addition of water for 16 h without enzyme addition increases the soluble organic nitrogen content in wheat bran from 14 to 43% (Arte et al. 2016). Roberts et al*.* extracted 66% of the protein from wheat bran at pH 4.5 followed by washing three times (pH 7), but in contrast to the present study, at a treatment time of 16 h and a temperature of 60 °C. The highest extraction rates were obtained with alkaline conditions (Roberts et al. 1985).

The calculated values of the metabolizable energy according to the formula of the GfE (Gesellschaft für Ernährungsphysiologie 2006) are based on analyzed results and table values on nutrient digestibilities of rye bran (Staudacher and Potthast 2014). The latter could deviate from the samples examined. Determination of the exact digestibilities is required in future studies using the classical methods on the target species to identify potential deviations from this estimated equation. Nevertheless, the determined energy values in the treated rye bran seem quite conceivable due to the low crude ash content and the associated high amount of organic residue. In addition, the consistent P reduction and a high nutritional profile of the feed produced across all process scales proofs the possibility to process larger quantities, enhancing productivity and optimizing the resource utilization. One of the key advantages is the valorization of biomass side streams generated in milling industry. The P reduction process introduced, paves the way for increased P utilization in animal feed, in line with regulatory requirements. The production of value-added feed material through depletion of phytate and utilization of phytate bound P in renewable resources contributes to a more sustainable and diversified P management.

### Comparison of physiochemical properties

The physiochemical properties of the feed material to which bran has been added, influence the sensory food quality. This also depends, among other things, on the water holding capacity (WHC). A high WHC results in a high swelling capacity. The particle size of the bran is also important, as it has been shown that this has an influence on the WHC (Petersson et al. 2013). In addition, the adherent P content is investigated, as it contributes to the total P content in the final product. Table 2 compares the equivalent circle diameters X_10_, X_50,_ and X_90_, the WHC, and the adherent P content for the P mobilization processes at different scales with the native substrate.

The alteration in particle size distribution is evident in the equivalent circle diameters, indicating a shift towards larger particle sizes post P mobilization. This trend is observed across all production scales, with the exception of the 400 L scale. The shift primarily results from the wash out of smaller particles during solid–liquid separation, and is reflected by the X_10_ values, which are consistently lower for all production scales compared to the native rye bran. However, the X_90_ values are increased due to agglomeration formation after drying, with not all agglomerates being appropriately dispersed by the measurement device. Despite this, the average particle size X_50_ does not seem to be clearly influenced by the P mobilization processes. This is explained by the fact that, in addition to washout and agglomerate formation, the stirrer and the soaking in liquid also affect particle distribution. The soaking, in particular, has an impact on the cell wall (Petersson et al. 2013), which also enables phytate mass transfer (Feizollahi et al. 2021). However, there is no difference between water or buffer as a reaction medium. In addition, the stirrer has an abrasive effect on the cell wall, which has been weakened by the soaking process. Due to the significantly higher energy input and the stirrer geometry in the 400 L reaction, the particle distribution after P mobilization is smaller. As expected, the agitator has the greatest influence on the resulting particle distribution.

WHC of the initial substrate was found to be 2.8 g/g, which is in agreement with literature sources (Petersson et al. 2013; Laurikainen et al. 1998). However, after P mobilization WHC increased by a factor of 1.7 to 2.1. Consequently, the conditioned rye bran has a higher capacity to swell compared to the native substrate. This is a desirable effect and is correlated with the fiber content (Table 1). The higher the fiber content, the more material is available for network formation, resulting in an increased WHC (Petersson et al. 2013).

The adherent P content varies from 0.4 to 1 g_P_/kg_DM_. It is noteworthy that the adherent P content is lower in the large scale than in the small scale. After sedimentation, larger wash volumes were used in relation to biomass. Considering the adherent P content (Table 2) and the total P content after P mobilization (Table 1), half of the P adheres to the final product. For maximum P reduction, washing of the biomass is required. However, the remaining P is digestible after phytate depletion (Fig. 3) and is therefore suitable as phytate-depleted animal feed. In order to minimize process wastewater, washing of the biomass may not be necessary.

### Phosphorus recovery

The two precipitation products known from wastewater treatment, struvite [MgNH_4_PO_4_·6H_2_O] and calcium hydrogen phosphate [CaHPO_4_∙H_2_O], are viable options for P recovery due to their technical feasibility and applicability. Struvite can be used as an eco-fertilizer due to its slow release properties. It has the potential to reduce nutrient runoff and subsequent water body pollution, making it ideal for use in agriculture (González-Morales et al. 2021). Calcium phosphate [CaHPO_4_], on the other hand, is used in chemistry, biology, and agronomy (Labgairi et al. 2020), and is of higher value than struvite (Jupp et al. 2021). The aim is to show that, in principle, mobilized P can simply be recovered from the process wastewater by precipitation. P precipitation was performed using the process wastewater from the 400 L reaction to show feasibility on a larger scale. In fact, buffer salts can affect the precipitation. However, as tab water and pH titration is more economically and ecologically viable compared to a buffered system, and achieves comparable P reductions (Fig. 1), precipitation was not investigated for buffered systems. To our knowledge, neither struvite nor calcium hydrogen phosphate precipitation has been published for a side stream in feed material processing so far.

**Fig. 5:**
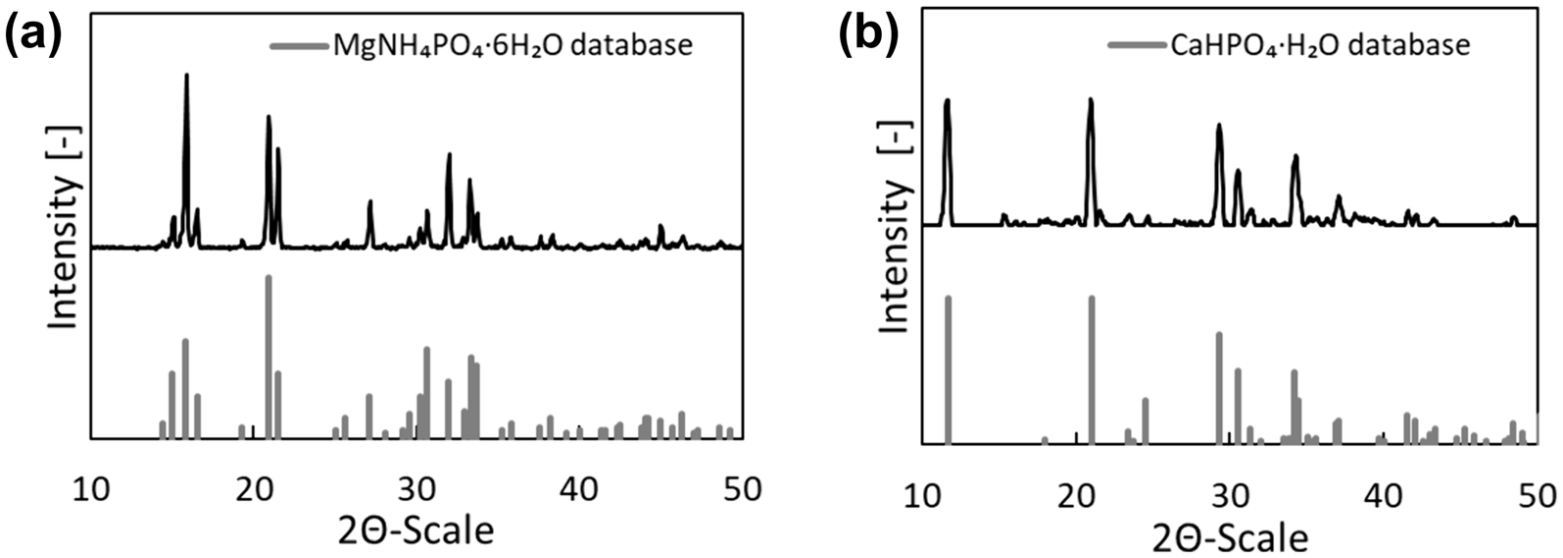
Diffraction diagram of **a** struvite and **b** calcium hydrogen phosphate precipitates measured at room temperature using Cu Kα radiation. Struvite precipitation was carried out for 1 h at pH 9 and room temperature, considering P in process wastewater and adding stoichiometric amounts of MgCl_2_ and NH_4_Cl. For CaHPO_4_ precipitation, a stoichiometric amount of Ca(OH)_2_ was used. The precipitation proceeded for 16 h. Struvite and CaHPO_4_ P recovery yields are 99% and 98% and XRD profiles score 82% and 87%, respectively

For the precipitation of struvite, a yield related to P of 99%, and for the precipitation of calcium hydrogen phosphate a yield of 98% was achieved. Both precipitates were confirmed by XRD analysis (Fig. 5). The results were compared with standards to determine how well their XRD profiles match. A higher score indicates a more appropriate match. According to the results, scores of 82% and 87% are found for struvite and calcium hydrogen phosphate, respectively. However, Fig. 5 shows that other phases and species may exist, suggesting some impurities. Relating the theoretical mass to the mass actually obtained gives purities of 80% and 86% for struvite and calcium hydrogen phosphate, respectively, which are in agreement with the data obtained by XRD. According to Gonzáles-Morales et al., who studied struvite precipitation in aerobic digest, these impurities can result from suspended solids (González-Morales et al. 2021). Ions in solution can also have an effect, especially Ca^2+^ on struvite precipitation (Li et al. 2016). Therefore, dilution of the process wastewater to reduce the concentration of interfering ions could increase the purity. In addition, more suitable process conditions, in terms of temperature and pH, can lead to higher purities of both products. It has been shown, that struvite precipitation at 30 °C is favorable (González-Morales et al. 2021; Hutnik et al. 2013; Jupp et al. 2021). However, considering the large-scale application and therefore prioritizing energy efficiency, minimizing costs and promoting environmental sustainability, the temperature was not controlled. Further downstream processing, e.g. vacuum filtration using appropriate wash solutions, could also achieve higher purities.

We demonstrate that P precipitation as struvite and calcium phosphate from the process wastewater from the enzymatic P mobilization process is a feasible P recovery strategy. As comparable P reductions in rye bran were achieved across all scales investigated, the concentration of mobilized P in the process wastewater is almost identical. Since precipitation is an effective P recovery technique for the largest scale presented, it can be assumed that precipitation is effective across scales.

## Conclusion

The successful implementation of the biotechnological P mobilization of feed material prior to feeding emphasize the production of P-depleted feed and the recovery of P for application in food, feed and as fertilizer. Our study demonstrates that the enzymatic P mobilization in bran can be scaled up (up to 400 L) leading to a complete conversion of InsP_6_ (P depletion of 91.2%) and a feed with a high nutritional profile. Phosphorus recovery from renewable resources is a step towards a better management of natural resources in a circular bioeconomy.

### Supplementary Information

Supplementary Material 1.

## Data Availability

All data generated or analysed during this study are included in this published article and its supplementary information files.

## Abbreviations

DM: Dry matter
HPIC: High Performance Ion chromatography
P: Phosphorus
PolyP: Polyphosphate
Bio-PolyP: Bio-based polyphosphate
InsP_1_: Inositol-monophosphate
InsP_2_: Inositol-diphosphate
InsP_3_: Inositol-triphosphate
InsP_4_: Inositol-tetraphosphate
InsP_5_: Inositol-pentaphosphate
InsP_6_: Inositol-hexaphosphate
WHC: Water holding capacity
rXT phy: rPhy XT52 phytase
DC phy: Phytase from *Debaryomyces castellii*
