# Supplementary material for: An up-scaled biotechnological approach for phosphorus-depleted rye bran as animal feed
Source: Bioresour Bioprocess. 2024 May 13;11(1):49. doi: 10.1186/s40643-024-00765-5 (PMC11091026; doi:10.1186/s40643-024-00765-5)
Supplement: Supplementary file 1 — Supplementary Material 1. [file 40643_2024_765_MOESM1_ESM.pdf]

Supplementary Information

An Up-Scaled Biotechnological Approach for Phosphorus-Depleted Rye Bran as Animal Feed

Niklas Widderich[1#], Johanna Stotz[2], Florian Lohkamp[3], Christian Visscher[3], Ulrich Schwaneberg[2], Andreas Liese[1], Paul Bubenheim[1*], Anna Joelle Ruff[2*]

[1] Institute of Technical Biocatalysis, Hamburg University of Technology, Germany, [2] Institute of Biotechnology, RWTH Aachen University, Germany, [3] Institute for Animal Nutrition, University of Veterinary Medicine Hanover, Foundation, Hanover, Germany

Table S1: In total 42 enzymatic P mobilization reactions were performed. The total P content, the obtained P reduction of the treated bran as well as the detailed reaction parameters are listed.

Reaction Nr.	Rye bran [kg]	Reaction medium		Reaction vessel and applied conditions						P Content				Rye bran used in P-mobilization reaction		P-reduction [%]
		Volume [L]	Medium	Type and size	Temperature	Stirring	Time	PH Titration	Na [g/kg uS]	P [g/kg uS]	TS [g/kg]	P [g/kg DM]	P [g/kg DM]	Batch Nr.	P [g/kg DM] native bran	
0	Batch 0	Native rye bran without treatment							0.022	11.65	901.5	12.92	**12.70** ± 0.91			
	Batch 00									12.42	908.00	13.67				
	Batch 1									10.48	901	11.63				
	Batch 2									10.56	907	11.64				
	Batch 3									11.68	917.5	12.73				
	Batch 4									12.38	909	13.61				
3a	0.1 kg	0.7 L	50 mM NaOAc, pH 5.0	5 L Beaker	26°C	Shaking 200 rpm	20h	no		1.68				4		
										1.77						
										1.28				3		
										0.72						
										1.61	955.0	1.68	**1.67** ± 0.03	4	13.61	**87.6**
										1.61	952.5	1.69				**87.5**
										1.57	956.5	1.64				**87.9**
3b					37°C					1.02				4		
										1.59						
										0.6				3		
										0.98	948.0	1.03	**1.17**	4	13.61	**92.4**
										1.50	953.5	1.57				**88.5**

										0.86	950.5	0.90	± 0.35			**93.4**
4a	0.1 kg	0.7 L	Deionized water	5 L Beaker	26°C	Shaking 200 rpm	20h	no		1.07				3		
										1.47				4	13.61	
										2.71						
										1.39	954.5	1.46	**1.42**	4	13.61	**89.3**
										1.15	957.0	1.20	±			**91.2**
										1.51	948.0	1.59	0.20			**88.3**
4b					37°C					0.72				**3**		
										1.28				**4**		
										1.16						
												0.90	**1.47**	**4**	13.61	**93.4**
											957.0					
										0.86						
										1.67			±			**87.1**
											955.0	1.75	0.49			
										1.68	955.5	1.76				**87**
5	0.2 kg	1.2 L	50 mM NaOAc, pH 5.0	Bioreactor 2.5 L	>37°C not controled	700 rpm	22h	off		1.71				**2**		
6	0.2 kg	1.2 L	50 mM NaOAc, pH 5.0	Bioreactor 2.5 L	37 °C	700 rpm	22h	pH 4.4 with HCl (2M) und NaOH		1.22	929.5	1.31	**1.23**	**3**	12.73	**89.7**
										0.64	929.5	0.69	±			**94.6**
							18 h			1.41	964.5	1.46	0.37	**4**	13.61	**89.3**
										1.42	967.5	1.47				**89.2**
7a	0.2 kg	1.2 L	Tap water	Bioreactor 2.5 L	37 °C	700 rpm	22h	pH 4.4 with HCl (2M) und NaOH		2.68	929.5	2.88	**2.72**	**3**	12.73	**77.4**
													±			**78.9**
										2.38	929.5	2.56	0.23			
7b			Deionized Water				18h			1.10	965.5	1.14	**1.11**	**4**	13.61	**91.6**
										1.04	967.0	1.08	±			**92**
													0.04			
8	8.65 kg	65 L	Deionized Water pH 4.4	Bioreactor 100 L	37°C	500 rpm	23h	pH 4.4 mit HCl. pH at t23h = 2.2	0.051	1.49				3		
										1.47						
										1.37	970.5	1.41	**1.41**	3	12.73	**88.9**
9	8.64 kg	65 L	Deionized Water			500 rpm	6h	pH 4.4 with HCl	0.29	1.08				3		
										1.14				3		

			pH 4.4					(2M) und NaOH		1.46	969.5	1.50	**1.50**	3	12.73	**88.2**
10	7.9 kg	65 L	Deionized Water pH 4.4	Bioreactor 100 L	37°C	500 rpm	6h	pH 4.4 with HCl (2M) und NaOH	0.032	1.11<br>1.07	941.0<br>976.5	1.18<br>1.09	**1.13** ± 0.06	4	13.61	**91.3**<br>**92**
11	9.1 kg	65 L	Deionized Water pH 4.4				6h		0.47	1.09<br>0.9	977<br>939.0	1.12<br>0.96	**1.04** ± 0.11	4	13.61	**91.8**<br>**92.9**
12	3 kg	20 L	Tap water	Vessel 32 L	RT - 22°C	no	20h	no		1.35	966.0	1.40	**1.40**	4	13.61	**89.7**
13	37.1 kg	248 L	Tap water	Vessel 400 L	29°C	yes	20 h	5M NaOH 5M HCl		1.25	979.5	1.28	**1.28**	3	12.73	**89.9**

HPIC chromatograms of InsP6 and phytate references

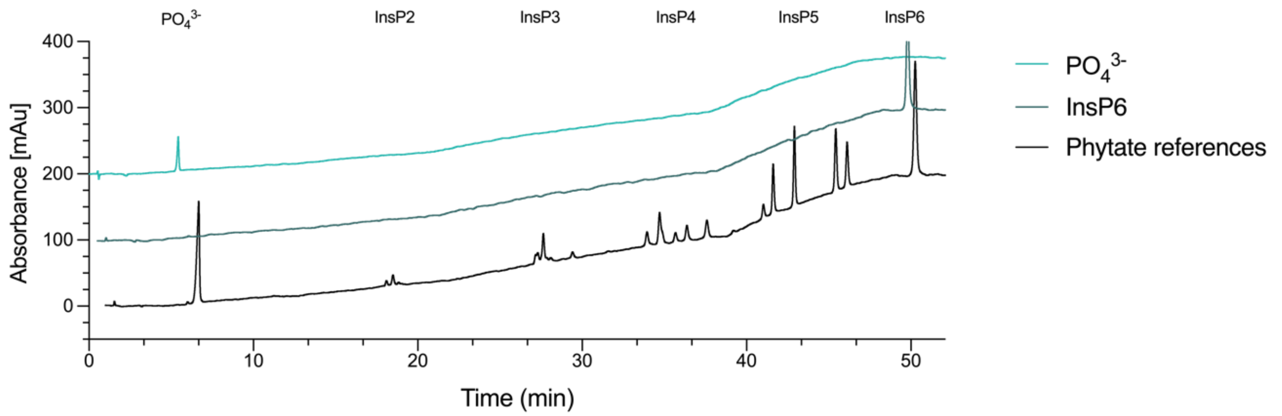

Figure S1: HPIC chromatograms of InsP6 and PO_4^{3-}standards and the phytate references standard, comprising a mixture of different isomers of InsP2-InsP6

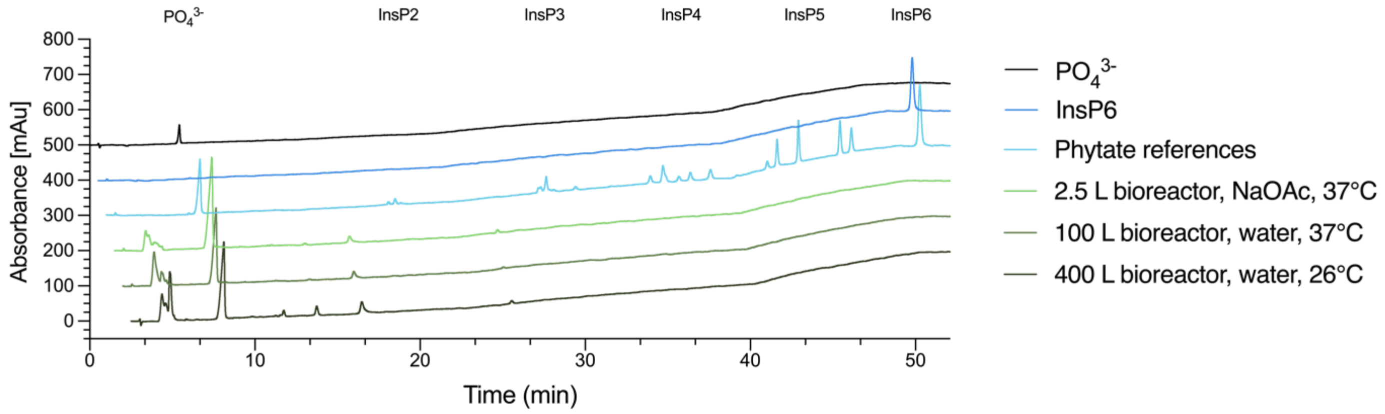

Figure S2: HPIC analysis of InsP6 and PO_4^{3-} in HCl-extracted rye bran after the P mobilization process comparing different reactor sizes. HPIC chromatogram extract from 0 to 50 min including single standards for InsP6 and PO_4^{3-}
